# The exploration of contrasting pathways in Triple Negative Breast Cancer (TNBC)

**DOI:** 10.1186/s12885-017-3939-4

**Published:** 2018-01-04

**Authors:** Shavira Narrandes, Shujun Huang, Leigh Murphy, Wayne Xu

**Affiliations:** 1 Research Institute of Oncology and Hematology, CancerCare Manitoba & University of Manitoba, Winnipeg, Canada; 2 Department of Biochemistry and Medical Genetics, University of Manitoba, Winnipeg, Canada; 3 College of Pharmacy, University of Manitoba, Winnipeg, Canada

**Keywords:** Triple Negative Breast Cancer, Pathway, Drug target, FOXM1, PPARα

## Abstract

**Background:**

Triple Negative Breast Cancers (TNBCs) lack the appropriate targets for currently used breast cancer therapies, conferring an aggressive phenotype, more frequent relapse and poorer survival rates. The biological heterogeneity of TNBC complicates the clinical treatment further. We have explored and compared the biological pathways in TNBC and other subtypes of breast cancers, using an in silico approach and the hypothesis that two opposing effects (Yin and Yang) pathways in cancer cells determine the fate of cancer cells. Identifying breast subgroup specific components of these opposing pathways may aid in selecting potential therapeutic targets as well as further classifying the heterogeneous TNBC subtype.

**Methods:**

Gene expression and patient clinical data from The Cancer Genome Atlas (TCGA) and the Molecular Taxonomy of Breast Cancer International Consortium (METABRIC) were used for this study. Gene Set Enrichment Analysis (GSEA) was used to identify the more active pathways in cancer (Yin) than in normal and the more active pathways in normal (Yang) than in cancer. The clustering analysis was performed to compare pathways of TNBC with other types of breast cancers. The association of pathway classified TNBC sub-groups to clinical outcomes was tested using Cox regression model.

**Results:**

Among 4729 curated canonical pathways in GSEA database, 133 Yin pathways (FDR < 0.05) and 71 Yang pathways (*p*-value <0.05) were discovered in TNBC. The FOXM1 is the top Yin pathway while PPARα is the top Yang pathway in TNBC. The TNBC and other types of breast cancers showed different pathways enrichment significance profiles. Using top Yin and Yang pathways as classifier, the TNBC can be further subtyped into six sub-groups each having different clinical outcomes.

**Conclusion:**

We first reported that the FOMX1 pathway is the most upregulated and the PPARα pathway is the most downregulated pathway in TNBC. These two pathways could be simultaneously targeted in further studies. Also the pathway classifier we performed in this study provided insight into the TNBC heterogeneity.

**Electronic supplementary material:**

The online version of this article (10.1186/s12885-017-3939-4) contains supplementary material, which is available to authorized users.

## Background

Breast cancer is the most commonly diagnosed cancer and leading cause of cancer-related deaths in women. In the US, it is the second-most common cause for cancer-related death in women, just behind lung cancer, with the expectation that 231,840 new cases will be diagnosed with 40,290 deaths in 2015 [1]. While breast cancer is typically referred to as a single disease, human breast tumors comprise heterogeneous and diverse groups, with patients in the same stage of disease varying in morphologies, treatments, treatment responses and overall outcomes [2]. With the advent of gene expression profiling technologies, researchers have been able to dissect the genetic and phenotypic variability among tumors and differentiate breast cancer into four molecular subtypes based on the presence or absence of the estrogen and/or progesterone hormone receptors (HR) and overexpression of the human epidermal growth factor 2 (HER2) protein: luminal A (HR+/HER2-), luminal B (HR+/HER2+), HER2-enriched (HR-/HER2+) and basal-like (HR-/HER2-) [2–5]. These groups are determined through the analysis of biological markers, which can provide diagnostic, prognostic and therapeutic response information about a certain cancer and are important in the early detection, diagnosis and treatment to improve patient outcome [6, 7]. Of the one million breast cancer cases annually diagnosed around the world, approximately 15–20%, or 170,000, of the cases will be of the Triple-Negative Breast Cancer (TNBC) subgroup [8–10]. Similar to breast cancers as a general group, TNBCs exhibit a disparity among racial groups, with premenopausal African and African American women demonstrating higher rates of diagnosis. Younger women, as well as Hispanic and non-Hispanic women of lower socioeconomic statuses, are also more frequently diagnosed with aggressive TNBCs [1, 9, 10]. Other risk factors include increased parity, younger age at first pregnancy, shorter period of breast feeding and higher hip-to-waist ratio [8].

Despite the widespread use of standard chemotherapy such as Paclitaxel (Taxol) or the combination of taxanes and genotoxic drugs, TNBCs lack the appropriate targets for the commonly used targeted breast cancer therapies, conferring an aggressive phenotype and poorer survival rate to the disease [8–12]. For example, Tamoxifen, which was originally used to treat all breast cancers, is now known to be effective against tumors expressing hormone receptors (ERs and PRs), while Trastuzumab therapy is used to treat patients presenting an over-amplification of HER2 [13]. Due to the lack of targeted therapies, TNBC patients have a poorer prognosis with more frequent relapse, distant recurrence and higher proliferation rates than other subtypes of breast cancer patients [8, 10–12].

Currently, many researchers are analyzing the dysfunctional pathways unique to TNBC in order to identify possible gene targets and develop drug therapies [14–17]. Although a couple of drugs are currently in undergoing clinical trials, the biology behind TNBC is still largely unknown. It is known that the TNBC represents distinct heterogeneity which complicates clinical treatment strategies. Further classification of TNBC may help in achieving better clinical outcome through. Currently, TNBC can be separated into distinct subtypes with gene expression profiling. Six subtypes have been reported with unique gene expression and ontologies: basal-like 1 (BL1), basal-like 2 (BL2), immunomodulatory (IM), mesenchymal (M), mesenchymal stem-like (MSL) and luminal androgen receptor (LAR) [18]. Masuda et al. [19] determined seven subtypes. In this study, we explored the pathways that are upregulated and downregulated in TNBC with respect to normal breast tissue samples. We hypothesized these up- and down-regulated pathways represent two opposing effects (Yin and Yang) that determine the cancer outcome [20–22]. These Yin and Yang pathways could help identify potential therapeutic targets for TNBC. They can be also used to build pathway classifiers in which the Yin and Yang pathways present a strong contrast pathway profile together. The TNBC subtypes classified by Yin and Yang pathways would aid in the personalized therapy for TNBC.

## Methods

### Gene expression data

The Cancer Genome Atlas (TCGA) uses genome analysis technologies, such as large-scale genome sequencing, to aid in the understanding of the molecular basis of cancer [23]. The mRNA (RNASeqV2) and clinical data were downloaded for 1085 patients with breast invasive carcinoma who had received pharmacological treatment (hormone therapy), chemotherapy, hormone and chemotherapy, an unknown treatment, or no treatment. Cases, which were either ER or PR or HER2 positive, were excluded such that 114 patients with TNBC remained.

For classifier comparison, we downloaded gene expression raw data files (.cel) of seven data sets from NCBI GEO database (GSE5327, GSE5847, GSE12276, GSE16446, GSE18864, GSE19615, and GSE20194). The expression values were summarized and normalized by Robust multiarray analysis (RMA) [24]. The Molecular Taxonomy of Breast Cancer International Consortium (METABRIC) is a joint Canada-UK project with the purpose of analyzing the molecular signatures of a large number of well-annotated breast tumors to further classify the tumors into subtypes [25]. The clinical traits and gene expression data were analyzed for ER, PR, and HER2 information resulting in the identification of 126 TNBC cases. In addition, two more sets (GSE58812, GSE25066) and cell line data (GSE10890) were used for prognostic signature validation.

### GSEA for TNBC pathways analysis

The TCGA patient data were grouped into seven sub-groups based on three commonly used markers: Triple Negative (TN), ER^+^/PR^+^/HER2^−^ (Luminal A), ER^−^/PR^−^/HER2^+^ (HER2 enriched), ER^+^/NODE^−^ (early ER+), ER^+^/NODE^+^ (late ER+), ER^+^/PR^+^/HER2^−^/NODE^−^ (early Luminal A), and ER^+^/PR^+^/HER2^−^/NODE^+^ (late Luminal A). The gene expression values for the tumor and normal breast samples were then put through Gene Set Enrichment Analysis (GSEA) [26] to generate an output of pathways that are upregulated and downregulated in each of these subtypes of breast cancer. Tests were run against the 4729 curated canonical pathways. The Yin (upregulated) pathways and Yang (downreguated) pathways were selected from these seven breast cancer sub-group analyses. The hierarchical cluster heat map using –log10 *p*-values or FDRs of pathways was used to compared the pathway differences among all seven breast cancer sub-groups.

### Pathway classifier

We hypothesize that the Yin and Yang pathways together present a contrast pathway profile for discrimination of cancer subgroups. We intended to develop classifiers for TNBC patients using the significant pathways derived from TNBC sample analysis. We first used all 204 pathways, including 133 Yin pathways (FDR < 0.05) and 71 Yang pathways (*p* < 0.05) (Additional files 1 and 2: Table S1 and S2). These FDR and *p*-value cutoff values were chosen because the default FDR < 0.25 [26] was too high for Yin pathway selection but too low for Yang pathway selection in the TNBC data analysis. The “Core” genes of these pathways were extracted and the weighted sum scores of each pathway were calculated. We first ordered all (n) the genes (xi) of the pathway according to their expression level, and then the weighted sum score = sum(xi* (n-i)/n). The TNBC samples were clustered by the pathways scores using Euclidean complete linkage. We then chose 16 pathways for pathway classifier testing. Among the top Yin pathways enriched in TNBC, most were involved in cell cycle regulation. We selected 8 top significant pathways that were involved in different stages of the cell cycle. The Yang pathways are the 8 most significantly downregulated pathways of the curated canonical pathways.

### Clinical outcome association study

We tested if the identified subgroups of TNBC have different clinical outcomes. The subgroups classified by multi-pathway classifier were tested against clinical information using Cox regression model. We used Partek Genomic Suite for these analyses. This test was to evaluate the clinical relevance of the pathway classifier.

We assume that the genes of the Yin and Yang pathways are both biologically and clinically relevant. Therefore we tested if the genes selected from all these pathways can be used to develop multigene signatures for TNBC prognosis. The “core” genes in the enriched pathway that contribute most to the gene enrichment results were selected. The “core” genes from the Yin pathways were the Yin genes and the “core” genes of the Yang pathways were the Yang genes. The Yin Yang gene expression mean ratio (YMR) signature [20–22] was tested using the TNBC samples of the TCGA and METABRIC datasets by the R package Survcomp.

## Results

### Pathways between TNBC and other subtypes of breast cancer

Among the 4729 curated canonical pathways, and using the TCGA dataset, 191 Yin pathways were discovered among the seven breast cancer groups where the FDR is less than 0.1 in at least one group and 176 Yang pathways where the *p*-value is less than 0.05 in at least one group (Additional files 1 and 2: Table S1 and S2.). We found the FOXM1 associated pathway is the top Yin upregulated pathway in TNBC but not in other subgroups of breast cancers. The PPARα associated pathway is the top listed Yang pathway TNBC but is also one of the pathways with similar significance shared with other breast cancer subtypes. Among those Yin pathways, the cell cycle related pathways are dominant in all types of breast cancers, including the FOXM1 pathway that interacts with cell cycle S, G2, and M phases, but are more significant in TNBC type than other types. Among the top Yang pathways, the GATA3 pathway showed unique significance in TNBC (Additional file 2: Table S2).

**Fig. 1:**
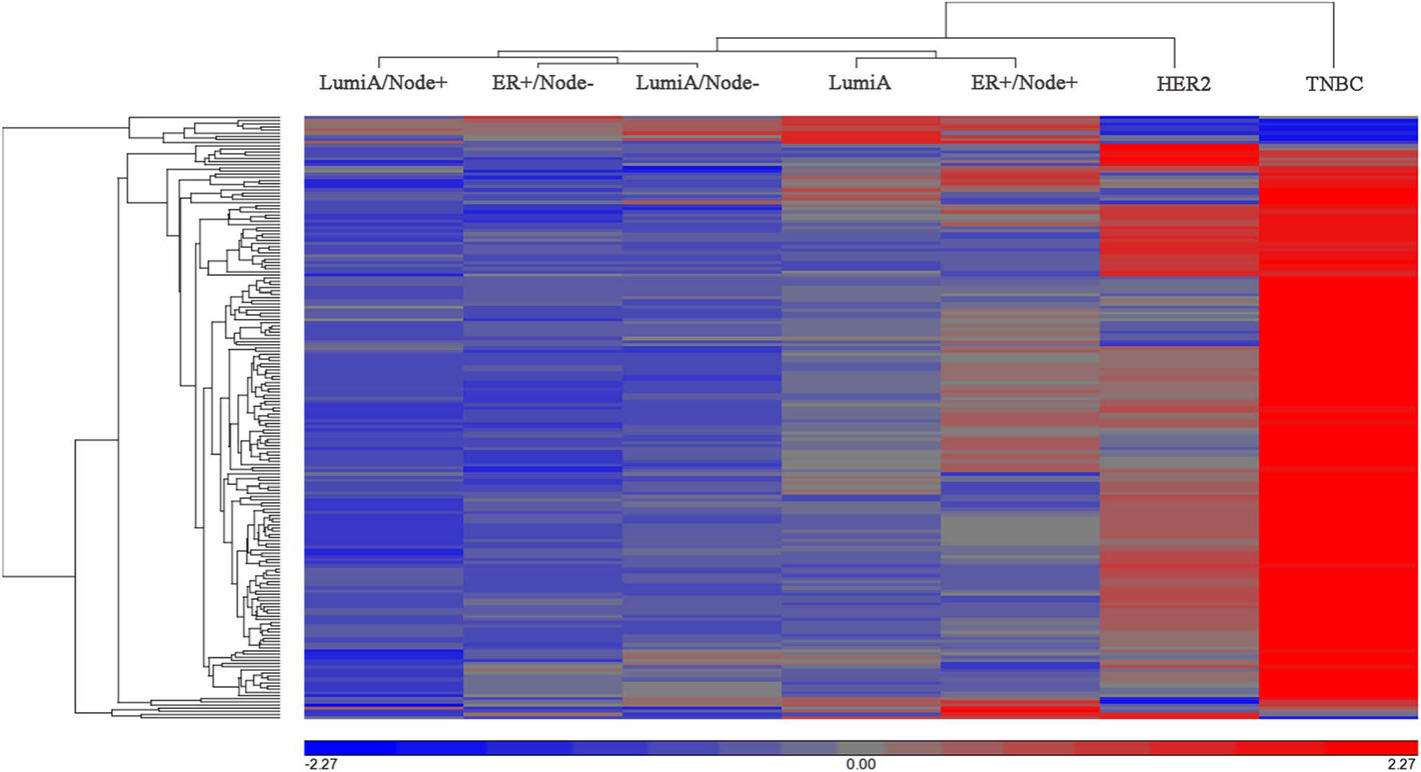
Yin pathway significant score profiling among 7 breast cancer subgroups using TCGA data. The significance values of 191 common Yin (upregulated) pathways (**rows**) were transformed into –log10 FDRs and standardized by mean of 0 and standard deviation of 1. The hierarchical Euclidean clustering with complete linkage was performed on all 7 breast cancer sub-groups (**columns**) using the pathway significant values

**Fig. 2:**
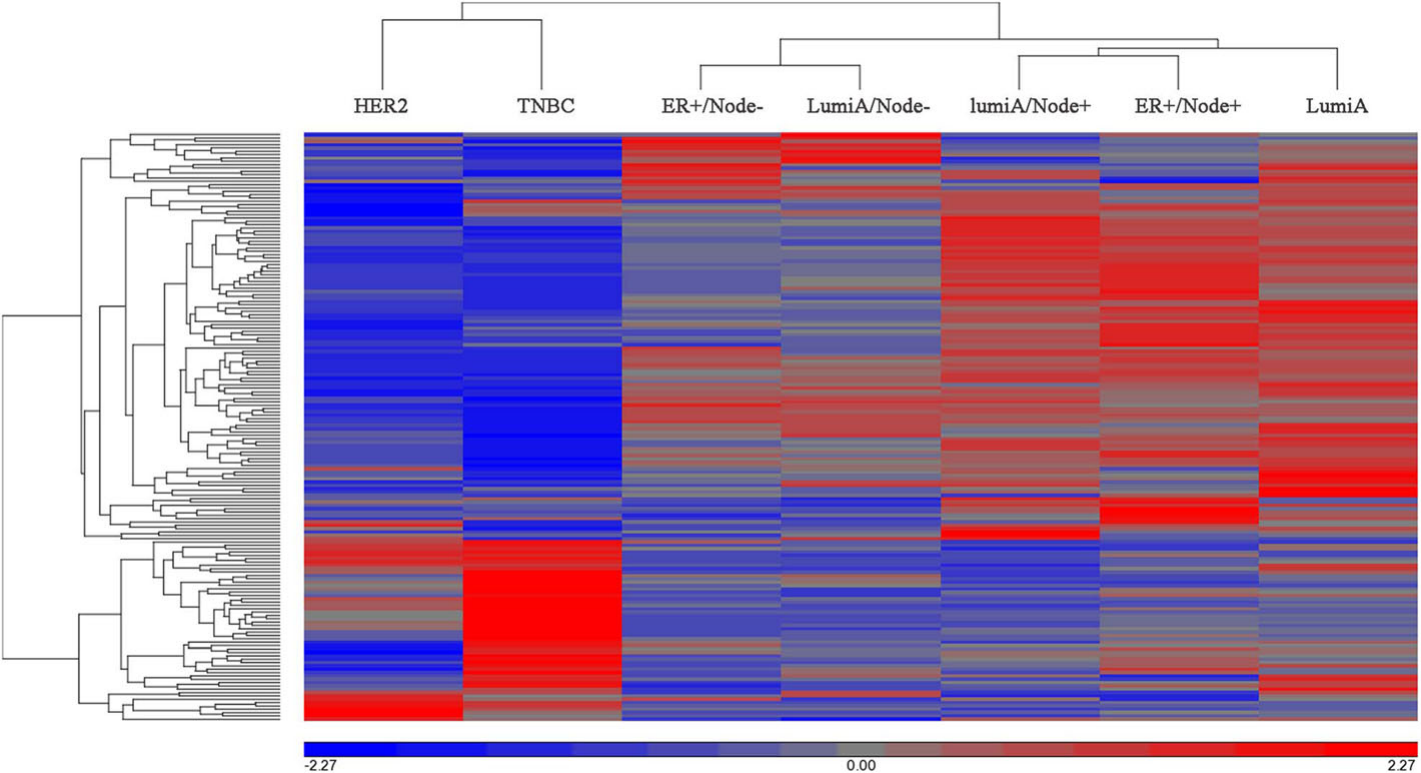
Yang pathway significant score profiling among 7 breast cancer subgroups using TCGA data. The significance values of 176 common Yang (downregulated) pathways (**rows**) were transformed into –log10 *p*-values and standardized by mean of 0 and standard deviation of 1. The hierarchical Euclidean clustering with complete linkage was performed on all 7 breast cancer sub-groups (**columns**) using the pathway significant values

The 2D complete linkage clustering showed the Yin pathway (Fig. 1) and Yang pathways (Fig. 2) significantly identified the seven breast cancer groups. The Yin pathway profile demonstrated that the TNBC is unique and distinct from the other six groups. In the Yang pathway profiling, TNBC were also classified as unique in most of the significant Yang pathways. However, using Yang pathways the TNBC seemed to share some similarity to the HER2 enriched subtype. These distinct patterns of the pathway enrichment significant scores were also shown among the intrinsic subtypes of breast cancers of TCGA data (Additional file 3: Figure S1 and S2).

### Pathway classifier for TNBC

For developing classifiers for TNBC, we chose the pathways from the TNBC pathway analysis above using the TCGA data set to classify the METABRIC cohort. Using all 133 significant Yin pathways (FDR < 0.05) and 71 Yang pathways (*p* < 0.05) we were able to classify the METABRIC TNBC into six subgroups based on the level three cluster branch (Additional file 3: Figure S3A). These six subgroups demonstrated strong contrasting Yin and Yang pathway score profiles. Different clinical outcomes were also found amongst these six subgroups, with cluster C1 having the highest 10 year overall survival time (>75%) and cluster C5 having the lowest OS time (35%) (Additional file 3: Figure S3B). These two clusters had highly contrasting Yin and Yang pathways scores (high score for all Yin pathways with low score for all Yang pathways, or high score for all Yang pathways with low score for all Yin pathways).

**Fig. 3:**
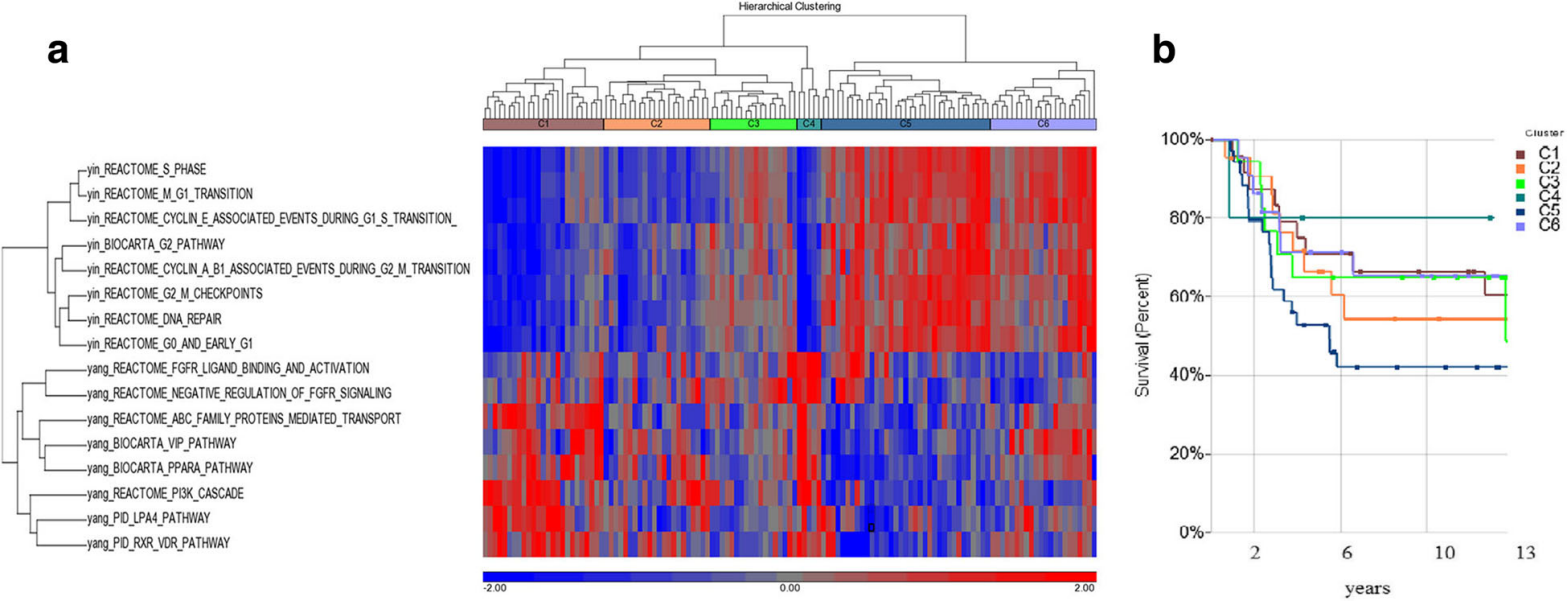
Yin Yang pathway classifier for METABRIC TNBCs. The weighted sum score was calculated for each of the 16 pathways (obtained from TCGA analysis) using the METABRIC dataset. The 126 TNBC samples of the METABRIC data set were clustered by the pathways scores using 2D Euclidean complete linkage (**a**). The clinical outcomes of the 6 clusters were evaluated by the Cox regression model using Partek Genomic Suite (**b**)

We further chose the top 8 Yin pathways that represent different stages of the cell cycle (for example, G0, G1, M-G1, G1-S, etc.) and the top 8 Yang pathways to build the pathway classifier. We applied this to the METABRIC TNBC cohort and as shown in Fig. 3a**,** the 16 pathways classifier on the METABRIC cohort, had an overall similar pathway score pattern to that found using the 204 pathway analysis on the METABRIC set (Additional file 3: Figure S3A), for example the C1, C2, C5, C6 in both sets. However, each of the patient clusters had different numbers of cases when the different classifiers (16 versus 204 pathways) were used (Fig. 3a versus Additional file 3: Figure S3A). In the 16-pathway classifier, the Cluster C5 still remained the highest risk group (Fig. 3b) because it had the highest contrast (high score for all Yin pathways with low score for all Yang pathways) of Yin and Yang pathway score profile (Fig. 3a). The cluster C6 had a higher OS rate than C5 (Fig. 3b) probably because C6 had higher pathway VIP and PPARα scores (higher intensity of red color) in the Yang pathway list (Fig. 3a). The cluster C4 had the lowest Yin and highest Yang contrast score profile, therefore showed the highest 10 year OS rate (80%). In the 16-pathway classifier, the cluster C1 did not show the highest OS rate, differing from the 204-pathway classifier, because this cluster was a mixed sub-cluster of high Yin pathway scores (Fig. 3a). We compared the 16 pathway classifier with a previously reported classification of seven TNBC subtypes using the same validation data sets of 201 samples [18]. Each of the six clusters identified using our 16-pathway classifier contains a variety of the previously defined subtypes [18]. This result suggested that these two approaches caught completely different features (Additional file 3: Figure S4).

### Pathway association to clinical outcome

**Fig. 4:**
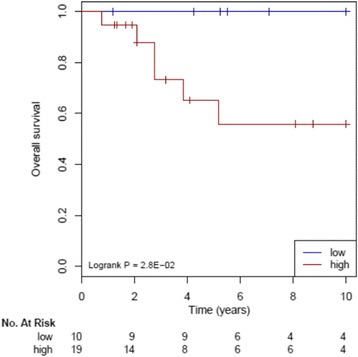
YMR signature built from the genes selected by Yin and Yang pathways. The “core” genes from the Yin pathways (133) were the Yin genes and the “core” genes of the Yang pathways (71) were the Yang genes. The Yin Yang gene expression mean ratio (YMR) signature [20] was tested using the untreated TNBC samples of the METABRIC dataset by the R package Survcomp

We tested if the core genes selected from the pathway analyses (using either 204 pathways, 16 pathways or 2 pathways i.e. FOXM1 and PPARα) can be used to build signatures for TNBC. One hundred and fourteen genes from the Yin (133) pathways and 66 genes from the list of Yang (71) pathways were then used in the YMR signature [20–22] and tested against the METABRIC dataset. All the 126 patients from the METABRIC dataset were separated into high risk and low risk groups using a median value of 1.00 and then survival curves over 10 years for the treated and untreated patients were generated. However, the survival curve graph for the treated and untreated patients in the low risk group did not show a significant stratification in survival outcomes. This is probably because chemotherapy disturbed the clinical association. When we used the 29 untreated TNBC patients, the YMR signature showed high risk and low risk group stratification significantly (log *P*-value of 2.8 × 10^−2^) though the group size is small (Fig. 4).

**Fig. 5:**
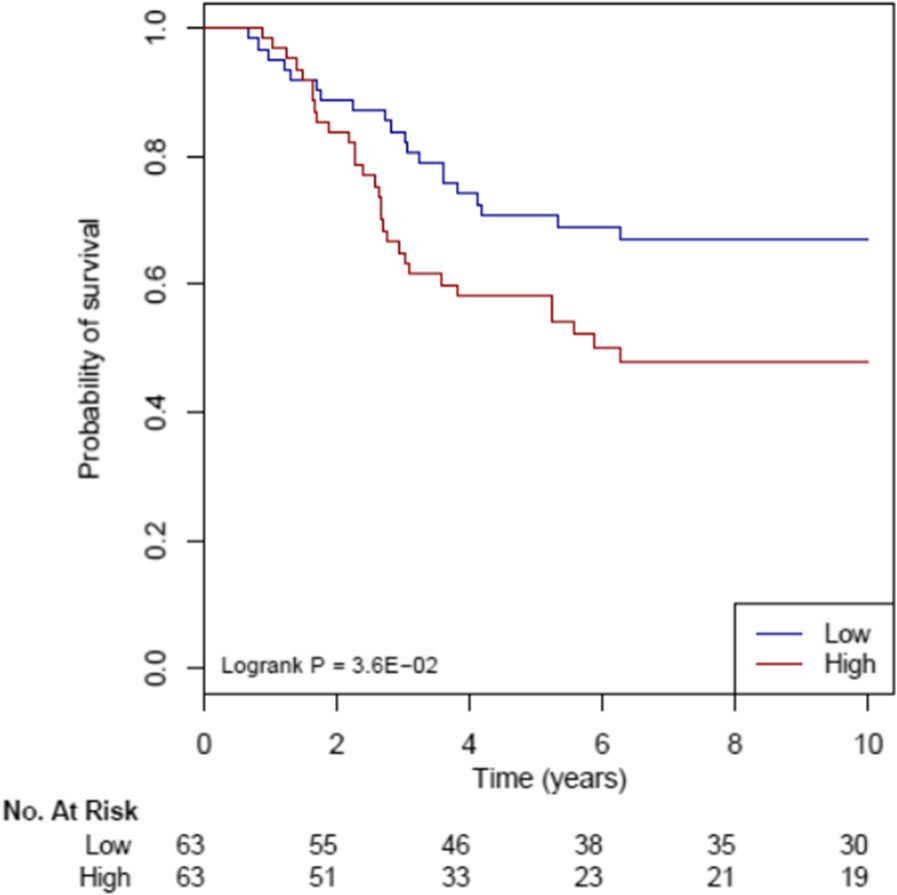
YMR signature built from FOXM1 and PPARα pathway genes. The YMR signature built using core genes of FOXM1 and PPARα pathways was tested using 126 METABRIC TNBC samples

We further tested if the YMR signature built using the top two FOXM1 and PPARα pathways only have prognostic value for TNBC. The two-pathway YMR significantly stratified the 126 METABRIC TNBC samples into low- and high-risk groups (Fig. 5). We examined the YMR score of the FOXM1 and PPARα pathways in breast cancer cell lines. As shown the YMR scores in ER-negative cell lines are higher than ER-positive cell lines with a moderate significant *p*-value (Additional file 3: Figure S5). However, this 2-pathway YMR score did not significantly stratify TNBC patients in another two independent cohorts (Additional file 3: Figure S6 and S7).

## Discussion

A number of the top pathways shown by GSEA to be upregulated in TNBC play a variety of roles in the mitotic cell cycle, cell division, and specific chromosomal processes. Of these pathways, the FOXM1, which is the top Yin pathway in TNBC but not in other breast cancer subtypes (i.e luminal, HER2 enriched), is listed as the most significant with a FDR of 0 (Additional file 1: Table S1). The FOXM1 includes Nek2, which is ranked first among all the genes from the gene sets characterized by GSEA (data not shown). Nek2, a member of the serine-threonine kinase family, is a cell cycle dependent protein kinase that has been shown to be upregulated in cancers such as lymphoma, cholangiocarcinoma, breast, prostate and cervical. Nek2 functions in the regulation of mitotic spindle formation, chromosome segregation, cell division, carcinogenesis, and the tumorigenic growth of breast cancer [27, 28]. It is especially known to play a role in the mitotic progression of cells where it prompts the separation of the centrosomes by centering itself on the centrosome and establishing a bipolar spindle [27]. This is noteworthy as chromosome instability is considered a common defect in cancer cells which may arise from malfunctions in cell division and the unequal separation of chromosomes to their respective daughter cells during mitosis [29].

PPARα is the top listed TNBC Yang pathway but is a pathway shared with the other breast cancer subtypes (Additional file 2: Table S2). Some of the key players in the PPARα pathway are the nuclear receptors from the family of peroxisome proliferator activator receptors (PPARs). They generally control cellular proliferation and differentiation, glucose and lipid metabolism, as well as adipocyte differentiation [30, 31]. PPARα ligands have been shown to induce cell cycle arrest at the G_1_ phase of the cell cycle to prompt the differentiation of liposarcoma and colon, prostate and breast cancer cells, conferring a less malignant phenotype to the cells. The induction of apoptosis through the PPARα pathway in the cells was accompanied by the activation of the NF-κB pathway, which functions in the inflammatory response, innate and adaptive immunity, and prevention of cells undergoing apoptosis following DNA damage [31, 32].

When we input all Yang pathway genes into Ingenuity Pathway Analysis system (IPA), again the top one is the PPARα/RXRα pathway with a *p*-value of 1.95 × 10^53^. The PPARα/RXRα pathway functions in both the cytoplasm and nucleus of cells. Retinoid X receptors (RXRs) are nuclear receptors that form heterodimers with retinoic acid receptors (RARs), which are ligand-regulated transcription factors, to control cell growth and survival. Retinoic acid binds to RARs to regulate processes such as development and cell proliferation, differentiation and apoptosis [33]. In the PPARα/RXRα pathway, PPARα and RXRα form a heterodimer which then binds to DNA to regulate gene transcription. From the IPA output, genes are then transcribed that function in fatty acid oxidation, lipoprotein metabolism, and anti-inflammation. There has been evidence that therapies combining PPARα and RXRα ligands in the treatment of breast cancer are effective [34]. Recently, there has been interest in the treatment of cancers using RAR and RXR modulators as it has been shown that the use of RAR modulation to treat acute promyelocytic leukemia has been successful. Therefore, the use of selective receptor modulators may help address the limitations of some drugs [35]. Selective agonist retinoids were studied in vitro to determine their effects on the proliferation and apoptosis of human breast cancer cells. As the PPARα/RXRα IPA pathway was constructed from the list of downregulated genes, it is possible that induction or amplification of PPARα/RXRα within TNBC cells may provide a better treatment for the disease.

Gene expression profiling has been used to separate TNBC into six subtypes with unique gene expression and ontologies: basal-like 1 (BL1), basal-like 2 (BL2), immunomodulatory (IM), mesenchymal (M), mesenchymal stem-like (MSL) and luminal androgen receptor (LAR) [18]. It was found that the *EGFR*, *VEGFR* and *FGFR* gene products were particularly amplified in TNBCs and serve as putative targets for drug therapies [18]. Although initially it was unclear as to the clinical significance of these subtypes, Masuda et al. [19] determined that a seven subtype classification, which includes an unstable (UNS) subtype, has the potential to aid in the development of innovative personalized medicine regimes for TNBC patients. More recently, though, Burstein et al. [36] analyzed the prognosis of TNBC subtypes and separated the disease into four groups: luminal androgen receptor (LAR), mesenchymal (MES), basal-like immunosuppressed (BLIS) and basal-like immune activated (BLIA) subtypes, with the worst prognosis conferred to BLIS and the most favourable to BLIA. Potential targets included androgen receptor and cell surface mucin (*MUC1*) for LAR, growth factor receptors such as platelet-derived growth factor (*PDGF*) receptor A for MES, immunosuppressing molecule (*VTCN1*) for BLIS and stat signal transduction molecules and cytokines for BLIA [36]. In this study, we used the pathway score profiles of the Yin and Yang pathways as a classifier for TNBC. The 6 subtypes of TNBC generated by our approach showed different pathway patterns and distinct clinical outcomes. We compared our 16-contrasting pathway classifier to the previous 7-subtype classifier using the same validation data [18]. We found that these two classifiers resulted in different classifications (Additional file 3: Figure S4). This is expected since we used the same pathway but different scores to differentiate subtypes while previous methods used gene expression profiling for clustering.

A different YMR signature model has demonstrated significance in stratifying TNBC into high- and low-risk groups though the cohort size is small. Due to the high level of molecular and clinical heterogeneity of TNBC, this range of significance suggested that the YMR built from the Yin Yang pathway genes or FOXM1, PPARα pathway genes has potential significance in some subgroups of TNBC. However, currently TNBC data are mostly collected from patients who underwent chemotherapy, which may disturb the prognosis detection we encountered in this study.

The limitation of this study is the validation of prognostic model of FOXM1 and PPARα pathways. In contrast to previous studies that purposely selected prognostic genes or pathways; we identified important pathways in TNBC tumor compared to normal and then tested their prognostic significance. We validated the 2-pathway prognostic model using the METABRIC data set. We attempted to validate our 2-pathway YMR model in other data sets (GSE28812, GSE25066), however although a similar pattern was found it did not achieve statistical significance. Therefore this is a limitation of our study. The reasons for this are unclear, although different treatments and the frequency of treated versus untreated cases in the cohorts may underlie the different results obtained. We must cautiously interpret the data where patients underwent therapy because therapy can alter prognosis or we were testing the treatment benefit. There is also a limitation in finding large sample size of TNBC without therapy treatment for our validation.

## Conclusion

Through the use of GSEA we explored the regulatory signaling pathways in TNBCs. The upregulated FOXM1 pathway and downregulated PPARα pathways were found to be the most significant in TNBC. Therefore, simultaneously targeting these two opposing pathways potentially could provide novel treatments options for some TNBC patients. The pathways can also be used as classifiers to subtype TNBC further for prognosis. The resulting TNBC subtypes exhibit different clinical outcomes, which supports the utility of our approach. This is a primary study using contrasting pathways for TNBC subtyping. Further study will focus on prognosis and treatment prediction signatures for each of these subgroups using more data sets.

## Additional files

Additional file 1: FDRs of 191 Yin . 133 Yin pathways were selected with PDF < 0.05 (XLS 73 kb)

Additional file 2: *P*-values of 176 Yang pathways among BC subtypes. 71 Yang pathways were selected with *p* < 0.05 (XLS 69 kb)

Additional file 3: Other results: **Figure S1.** Yin pathway significant score profiling among LumA, LumB, Her2, TNBC breast cancer subtype using TCGA data. **Figure S2.** Yang pathway significant score profiling among LumA, LumB, Her2, TNBC breast cancer subtype using TCGA data. **Figure S3.** Yin Yang pathway classifier for TNBCs. **Figure S4.** Pathway classifier comparison. **Figure S5.** YMR scores of FOXM1 and PPARa pathway among Breast caner cell lines. **Figure S6.** FOXM1 and PPARa YMR model for GSE58812 data set. **Figure S7.** FOXM1 and PPARa YMR model for GSE25066 data set. (PDF 1224 kb)

## Abbreviations

BL1: Basal-like 1
BL2: Basal-like 2
ER: Estrogen receptor
FOXM1: Forkhead Box M1
GSEA: Gene Set Enrichment Analysis
HER2: Hormone epidermal growth factor receptor 2
IM: Immunomodulatory
LAR: Luminal androgen receptor
M: Mesenchymal
METABRIC: the Molecular Taxonomy of Breast Cancer International Consortium
MSL: Mesenchymal stem-like
PPARα: Peroxisome proliferator-activated receptor
PR: Progesterone receptor
TCGA: The Cancer Genome Atlas
TNBC: Triple Negative Breast Cancers
YMR: Yin Yang gene expression mean ratio

## References

[1] DeSantis CE, Fedewa SA, Goding Sauer A, Kramer JL, Smith RA, Jemal A (2016). Breast cancer statistics, 2015: convergence of incidence rates between black and white women. CA Cancer J Clin.

[2] Blows FM, Driver KE, Schmidt MK, Broeks A, Van Leeuwen FE, Wesseling J (2010). Subtyping of breast cancer by immunohistochemistry to investigate a relationship between subtype and short and long term survival: a collaborative analysis of data for 10,159 cases from 12 studies. PLoS Med.

[3] Anderson WF, Rosenberg PS, Katki HA (2014). Tracking and evaluating molecular tumor markers with cancer registry data: HER2 and breast cancer. J Natl Cancer Inst.

[4] Barnard ME, Boeke CE, Tamimi RM (2015). Established breast cancer risk factors and risk of intrinsic tumor subtypes. Biochimica et Biophysica Acta (BBA)-Reviews on Cancer.

[5] Tamimi RM, Colditz GA, Hazra A, Baer HJ, Hankinson SE, Rosner B (2012). Traditional breast cancer risk factors in relation to molecular subtypes of breast cancer. Breast Cancer Res Treat.

[6] Hwa HL, Kuo WH, Chang LY, Wang MY, Tung TH, Chang KJ (2008). Prediction of breast cancer and lymph node metastatic status with tumour markers using logistic regression models. J Eval Clin Pract.

[7] Ludwig JA, Weinstein JN (2005). Biomarkers in cancer staging, prognosis and treatment selection. Nat Rev Cancer.

[8] Chacón RD, Costanzo MV (2010). Triple-negative breast cancer. Breast Cancer Res.

[9] Cleator S, Heller W, Coombes RC (2007). Triple-negative breast cancer: therapeutic options. Lancet Oncol.

[10] Ismail-Khan R, Bui MM (2010). A review of triple-negative breast cancer. Cancer Control.

[11] Liedtke C, Mazouni C, Hess KR, André F, Tordai A, Mejia JA (2008). Response to neoadjuvant therapy and long-term survival in patients with triple-negative breast cancer. J Clin Oncol.

[12] Yu K-D, Zhu R, Zhan M, Rodriguez AA, Yang W, Wong S (2013). Identification of prognosis-relevant subgroups in patients with chemoresistant triple-negative breast cancer. Clin Cancer Res.

[13] Dent R, Trudeau M, Pritchard KI, Hanna WM, Kahn HK, Sawka CA (2007). Triple-negative breast cancer: clinical features and patterns of recurrence. Clin Cancer Res.

[14] O'shaughnessy J, Osborne C, Pippen JE, Yoffe M, Patt D, Rocha C (2011). Iniparib plus chemotherapy in metastatic triple-negative breast cancer. N Engl J Med.

[15] O'Shaughnessy J, Schwartzberg L, Danso MA, Miller KD, Rugo HS, Neubauer M (2014). Phase III study of iniparib plus gemcitabine and carboplatin versus gemcitabine and carboplatin in patients with metastatic triple-negative breast cancer. J Clin Oncol.

[16] Rajamanickam S, Subbarayalu P, Timilsina S, Drake MT, Zhao Z, Chen HIH (2015). Imipramine blue: a novel NOX inhibitor as potent therapeutic agent to treat triple-negative breast cancers.

[17] Telli ML, Jensen KC, Vinayak S, Kurian AW, Lipson JA, Flaherty PJ (2015). Phase II study of gemcitabine, carboplatin, and iniparib as neoadjuvant therapy for triple-negative and BRCA1/2 mutation–associated breast cancer with assessment of a tumor-based measure of genomic instability: PrECOG 0105. J Clin Oncol.

[18] Lehmann BD, Bauer JA, Chen X, Sanders ME, Chakravarthy AB, Shyr Y (2011). Identification of human triple-negative breast cancer subtypes and preclinical models for selection of targeted therapies. J Clin Invest.

[19] Masuda H, Baggerly KA, Wang Y, Zhang Y, Gonzalez-Angulo AM, Meric-Bernstam F, et al. Differential pathologic complete response rates after neoadjuvant chemotherapy among molecular subtypes of triple-negative breast cancer. Journal of Clinical Oncology. 2013;31(no. 15_suppl):1005-1005.

[20] Xu W, Banerji S, Davie JR, Kassie F, Yee D, Kratzke R (2013). Yin Yang gene expression ratio signature for lung cancer prognosis. PLoS One.

[21] Xu W, Jia G, Cai N, Huang S, Davie JR, Pitz M (2017). A 16 yin Yang gene expression ratio signature for ER+/node-breast cancer. Int J Cancer.

[22] Xu W, Jia G, Davie JR, Murphy L, Kratzke R, Banerji S (2016). A 10-gene yin Yang expression ratio signature for stage IA and IB non–small cell lung cancer. J Thorac Oncol.

[23] Weinstein JN, Collisson EA, Mills GB, Shaw KRM, Ozenberger BA, Ellrott K (2013). The cancer genome atlas pan-cancer analysis project. Nat Genet.

[24] Irizarry RA, Hobbs B, Collin F, Beazer-Barclay YD, Antonellis KJ, Scherf U (2003). Exploration, normalization, and summaries of high density oligonucleotide array probe level data. Biostatistics.

[25] Curtis C, Shah SP, Chin S-F, Turashvili G, Rueda OM, Dunning MJ (2012). The genomic and transcriptomic architecture of 2,000 breast tumours reveals novel subgroups. Nature.

[26] Subramanian A, Tamayo P, Mootha VK, Mukherjee S, Ebert BL, Gillette MA (2005). Gene set enrichment analysis: a knowledge-based approach for interpreting genome-wide expression profiles. Proc Natl Acad Sci.

[27] Cappello P, Blaser H, Gorrini C, Lin D, Elia A, Wakeham A (2014). Role of Nek2 on centrosome duplication and aneuploidy in breast cancer cells. Oncogene.

[28] Tsunoda N, Kokuryo T, Oda K, Senga T, Yokoyama Y, Nagino M (2009). Nek2 as a novel molecular target for the treatment of breast carcinoma. Cancer Sci.

[29] Hayward DG, Fry AM (2006). Nek2 kinase in chromosome instability and cancer. Cancer Lett.

[30] Chinetti G, Lestavel S, Bocher V, Remaley AT, Neve B, Torra IP (2001). PPAR-α and PPAR-γ activators induce cholesterol removal from human macrophage foam cells through stimulation of the ABCA1 pathway. Nat Med.

[31] Lorincz A, Sukumar S (2006). Molecular links between obesity and breast cancer. Endocr Relat Cancer.

[32] Bonizzi G, Karin M (2004). The two NF-κB activation pathways and their role in innate and adaptive immunity. Trends Immunol.

[33] Bushue N, Wan Y-JY (2010). Retinoid pathway and cancer therapeutics. Adv Drug Deliv Rev.

[34] Crowe DL, Chandraratna RA (2004). A retinoid X receptor (RXR)-selective retinoid reveals that RXR-α is potentially a therapeutic target in breast cancer cell lines, and that it potentiates antiproliferative and apoptotic responses to peroxisome proliferator-activated receptor ligands. Breast Cancer Res.

[35] Altucci L, Leibowitz MD, Ogilvie KM, De Lera AR, Gronemeyer H (2007). RAR and RXR modulation in cancer and metabolic disease. Nat Rev Drug Discov.

[36] Burstein MD, Tsimelzon A, Poage GM, Covington KR, Contreras A, Fuqua SA (2015). Comprehensive genomic analysis identifies novel subtypes and targets of triple-negative breast cancer. Clin Cancer Res.

