# Supplementary material for: The exploration of contrasting pathways in Triple Negative Breast Cancer (TNBC)
Source: BMC Cancer. 2018 Jan 4;18:22. doi: 10.1186/s12885-017-3939-4 (PMC5753474; doi:10.1186/s12885-017-3939-4)
Supplement: Supplementary file 3 — Other results: Figure S1. Yin pathway significant score profiling among LumA, LumB, Her2, TNBC breast cancer subtype using TCGA data. Figure S2. Yang pathway significant score profiling among LumA, LumB, Her2, TNBC breast cancer subtype using TCGA data. Figure S3. Yin Yang pathway classifier for TNBCs. Figure S4. Pathway classifier comparison. Figure S5. YMR scores of FOXM1 and PPARa pathway among Breast caner cell lines. Figure S6. FOXM1 and PPARa YMR model for GSE58812 data set. Figure S7. FOXM1 and PPARa YMR model for GSE25066 data set. (PDF 1224 kb) [file 12885_2017_3939_MOESM3_ESM.pdf]

**Additional file 3**

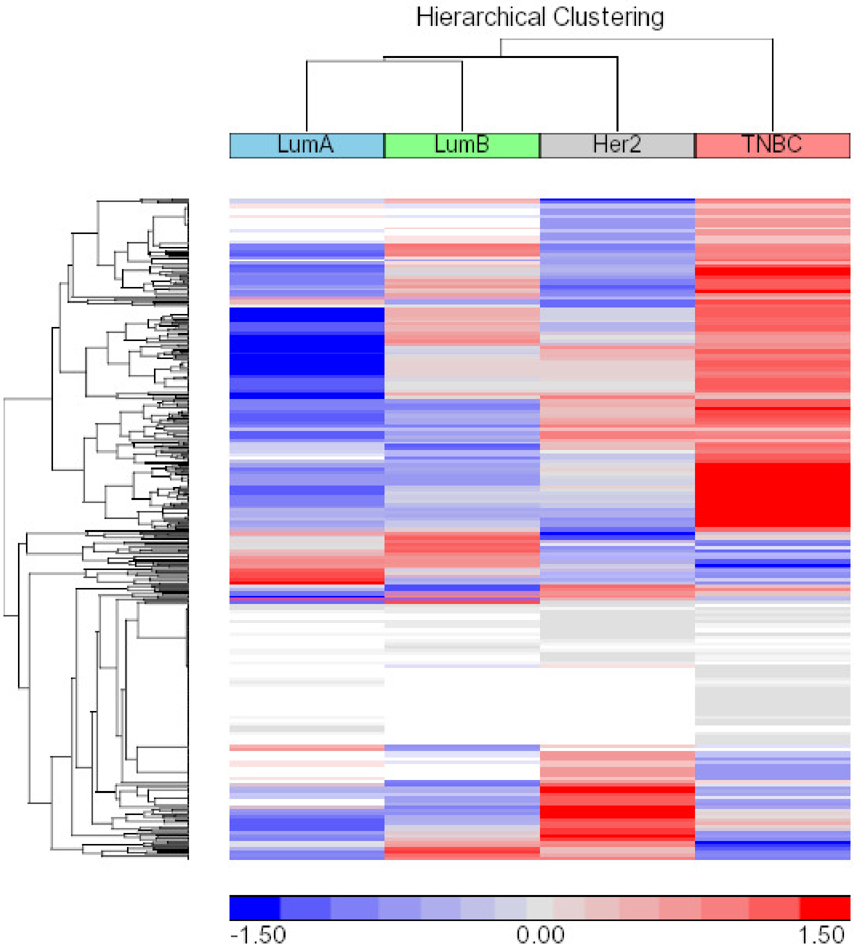


Fig. S1. **Yin pathway significant score profiling among LumA, LumB, Her2, TNBC breast cancer subtype using TCGA data.**

The significant values of Yin (upregulated) pathways (**rows, C2 canonical pathways v5.2**) were transformed into –log10 FDRs and standardized by mean of 0 and standard deviation of 1. The hierarchical Euclidean clustering with complete linkage was performed on breast cancer subtypes (**columns**) using the pathway significant values.

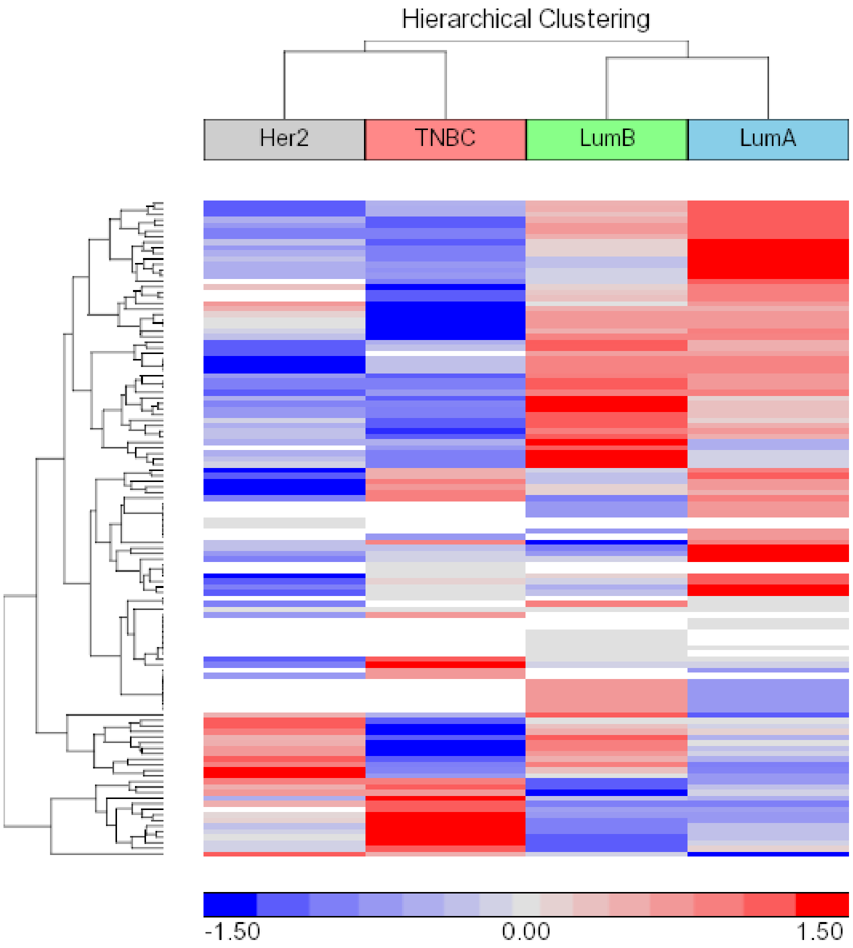


Fig. S2. **Yang pathway significant score profiling among LumA, LumB, Her2, TNBC breast cancer subtype using TCGA data.**

The significant values of Yang (upregulated) pathways (**rows, C2 biocarta pathways v5.2**) were transformed into –log10 FDRs and standardized by mean of 0 and standard deviation of 1. The hierarchical Euclidean clustering with complete linkage was performed on breast cancer subtypes (**columns**) using the pathway significant values.

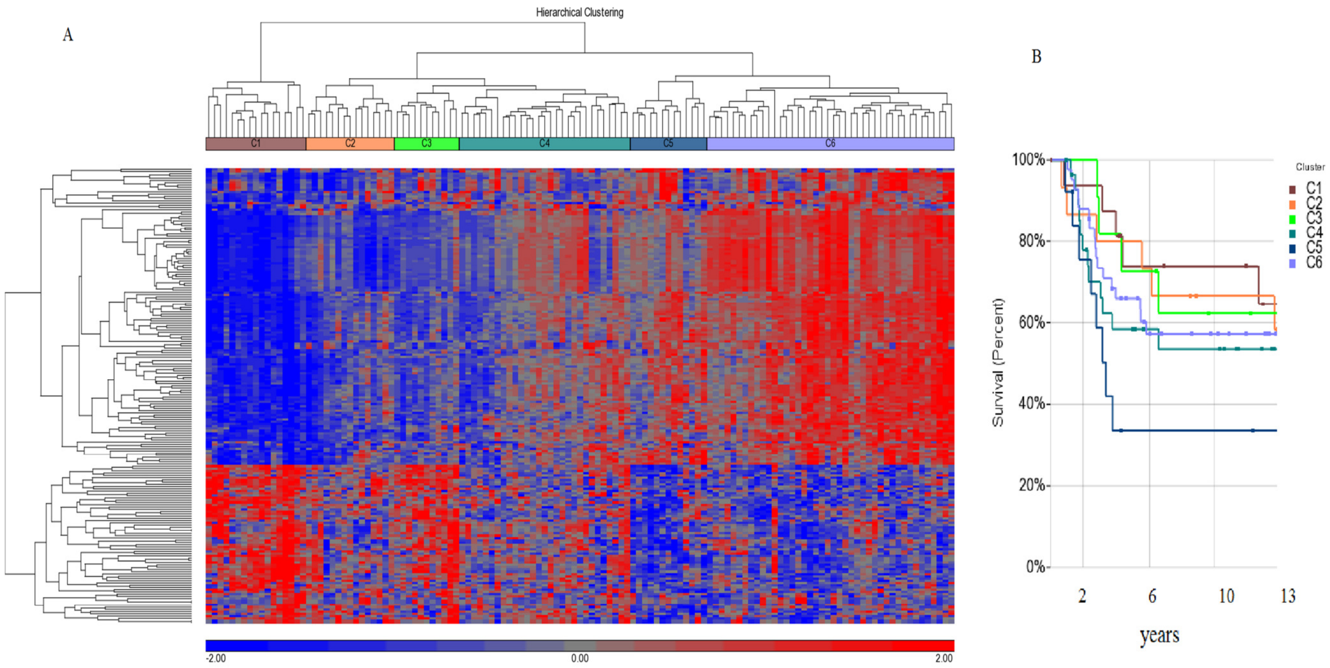


**Fig. S3 Yin Yang pathway classifier for TNBCs.**

The “core” genes selected from 133 Yin pathways (different cell cycle phage pathways) and 71 top Yang pathways. The weighted sum score was calculated for each of the 204 pathways. The 126 TNBC samples of the METABRIC data set were clustered by the pathways scores using 2D Euclidean complete linkage (A). The clinical outcomes of the 6 clusters were evaluated by the Cox regression model using Partek Genomic Suite.

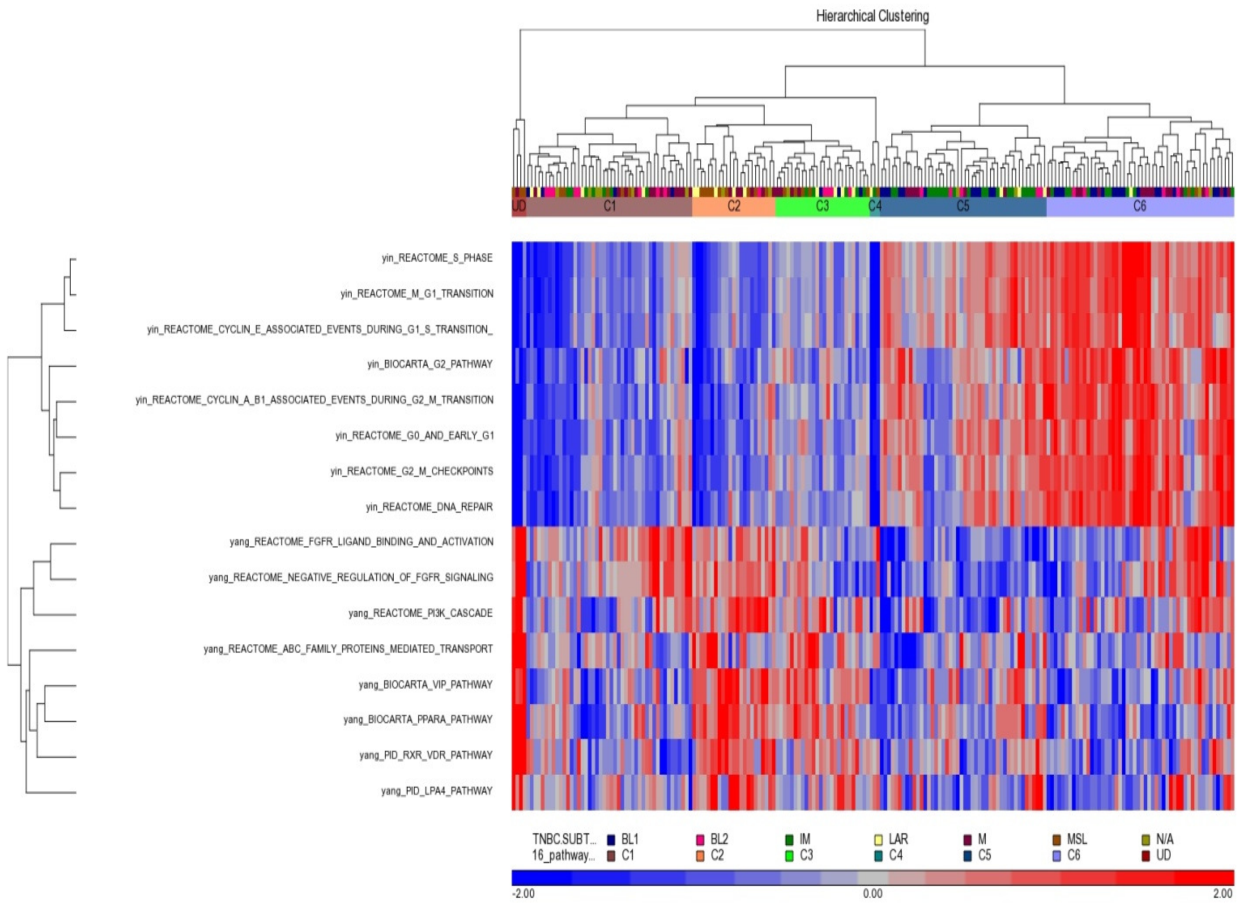


Fig. S4. **Pathway classifier comparison**. 16-pathway classifier was applied to the 201 TNBC samples that were subtyped by previous Lehmann method. Color boxes with C1 to C6 were clusters by 16 pathway scores. The color bars above represented previous 7 subtypes.

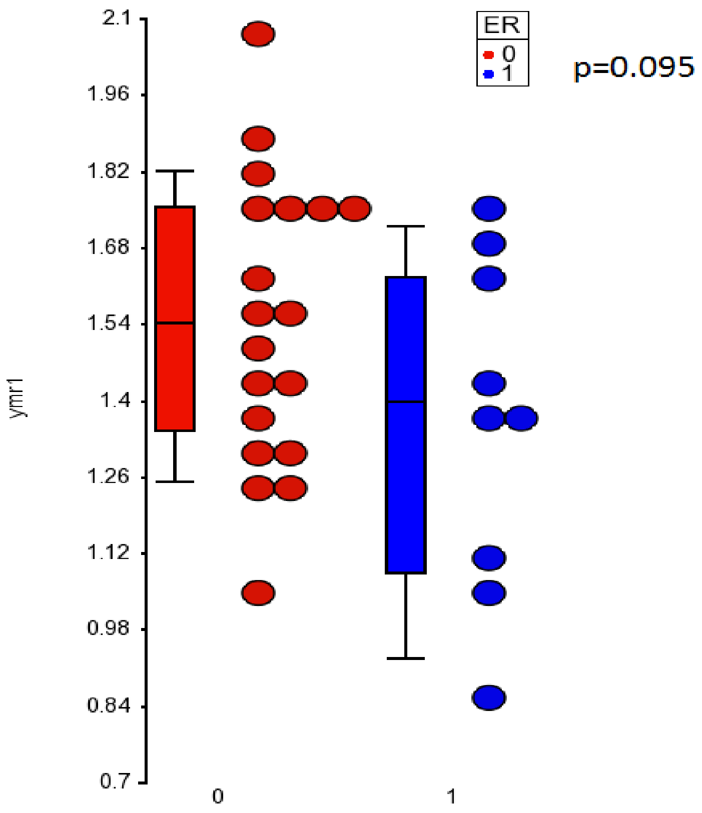


| Access | breast cancer cell_line | 2-pathway ymr | ER(+/1,-/0) |
|---|---|---|---|
| GSM276061 | BT474 | 1.400 | 1 |
| GSM276035 | MDAMB361.1 | 1.032 | 1 |
| GSM276025 | ZR75 | 1.085 | 1 |
| GSM276018 | HCC1428 | 1.679 | 1 |
| GSM276007 | MDAMB415 | 1.412 | 1 |
| GSM276000 | BT474EI | 0.824 | 1 |
| GSM275992 | T47D | 1.760 | 1 |
| GSM275979 | BT483 | 1.374 | 1 |
| GSM275978 | MCF7 | 1.626 | 1 |
| GSM276032 | UACC812 | 1.068 | 0 |
| GSM276028 | HCC38 | 1.446 | 0 |
| GSM276027 | HCC1569 | 1.748 | 0 |
| GSM276023 | HCC1143 | 1.792 | 0 |
| GSM276021 | HCC1500 | 1.247 | 0 |
| GSM276019 | HCC1954 | 1.763 | 0 |

| | | | |
|---|---|---|---|
| GSM276015 | SKBR31 | 1.282 | 0 |
| GSM276013 | HCC70 | 1.643 | 0 |
| GSM276009 | MDAMB468 | 1.779 | 0 |
| GSM276003 | MDAMB453 | 1.258 | 0 |
| GSM275998 | MDA361 | 1.398 | 0 |
| GSM275997 | HCC1937 | 1.738 | 0 |
| GSM275994 | MDA468 | 1.514 | 0 |
| GSM275993 | MDA231 | 1.562 | 0 |
| GSM275989 | MDA435 | 2.105 | 0 |
| GSM275986 | MDA436 | 1.543 | 0 |
| GSM275983 | SKBR3 | 1.293 | 0 |
| GSM275977 | HS578T | 1.433 | 0 |
| GSM275974 | BT549 | 1.853 | 0 |

**Fig. S5 YMR scores of FOXM1 and PPARa pathway among Breast caner cell lines (GSE10890).** 28 unique cell lines with known ER marker status were selected. The cell line marker information was from Neve RM, et al (Cancer Cell. 2006, 10(6): 515–527).

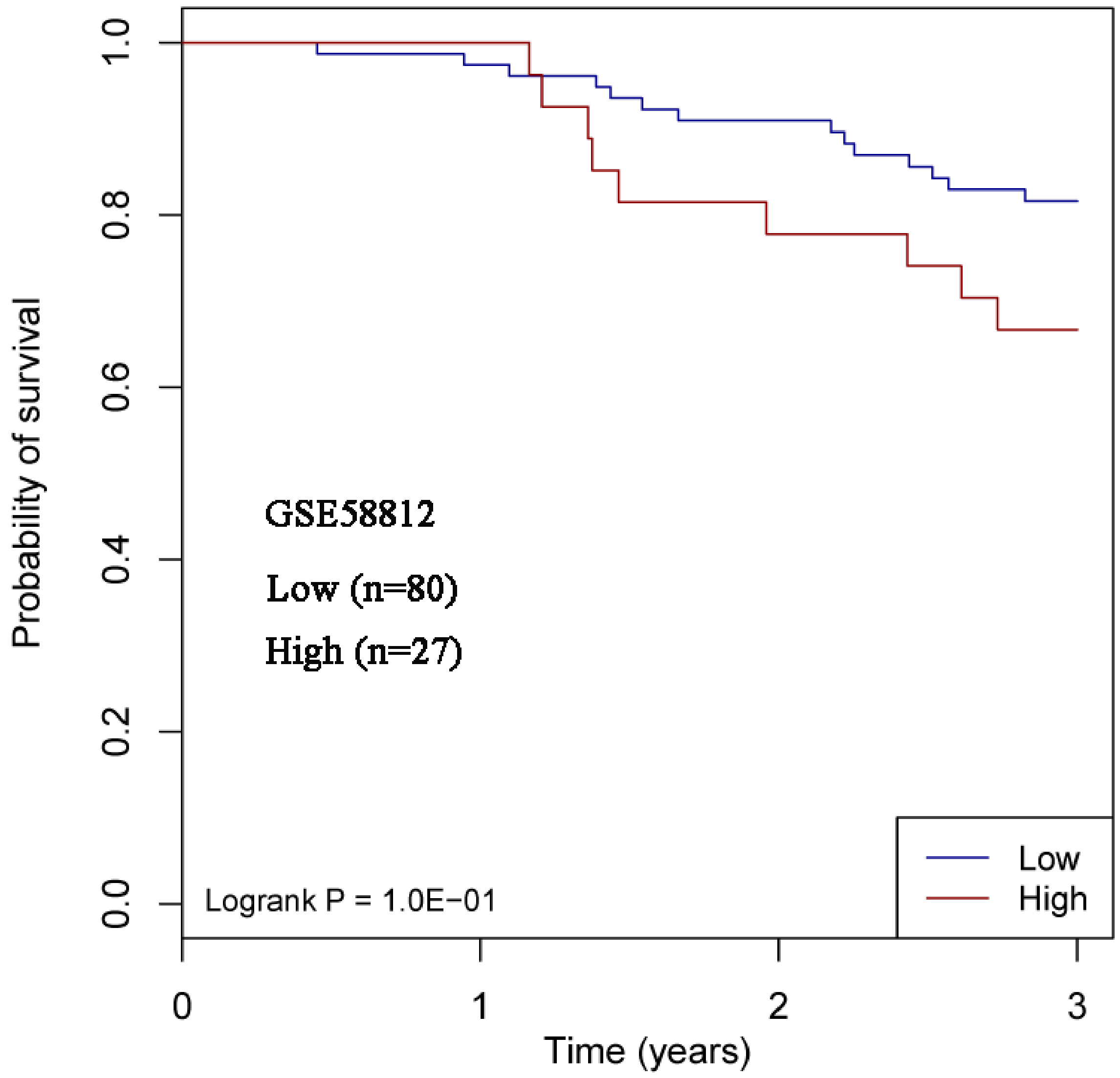


**Fig. S6. FOXM1 and PPARa YMR model for GSE58812 data set.** The patients were stratified into low and high risk but with no significance.

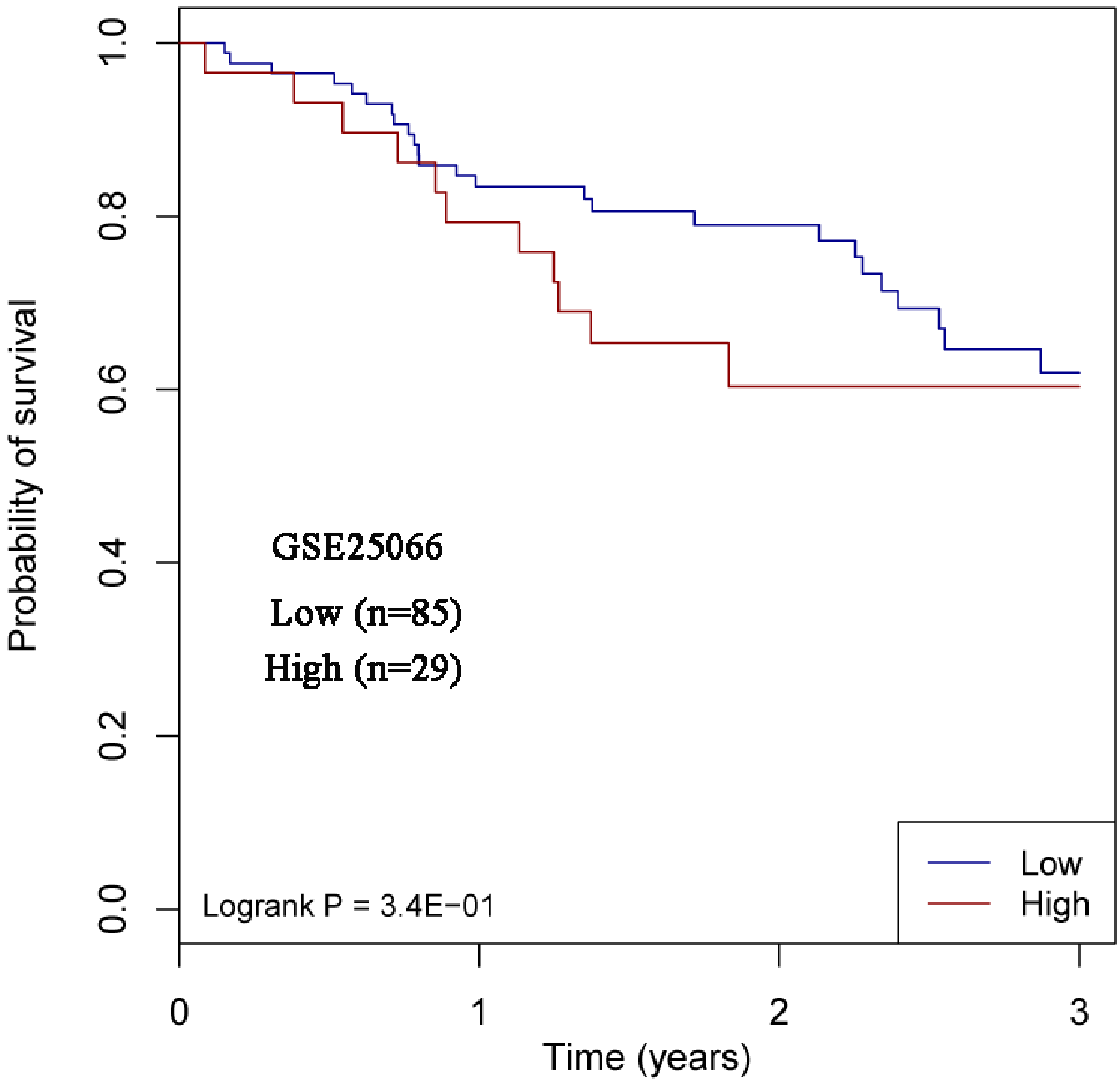


**Fig. S7. FOXM1 and PPARa YMR model for GSE25066 data set.** The patients were stratified into low and high risk but with no significance. 100% of patients underwent therapy.
